# Expanding access to veterinary clinical decision support in resource-limited settings: a scoping review of clinical decision support tools in medicine and antimicrobial stewardship

**DOI:** 10.3389/fvets.2024.1349188

**Published:** 2024-06-04

**Authors:** Havan Yusuf, Alison Hillman, Jan Arend Stegeman, Angus Cameron, Skye Badger

**Affiliations:** ^1^Ausvet, Fremantle, WA, Australia; ^2^Department of Farm Animal Health, Faculty of Veterinary Medicine, Utrecht University, Utrecht, Netherlands; ^3^Ausvet Europe, Lyon, France

**Keywords:** decision support tools, digital, diagnosis, treatment, antimicrobial stewardship

## Abstract

**Introduction:**

Digital clinical decision support (CDS) tools are of growing importance in supporting healthcare professionals in understanding complex clinical problems and arriving at decisions that improve patient outcomes. CDS tools are also increasingly used to improve antimicrobial stewardship (AMS) practices in healthcare settings. However, far fewer CDS tools are available in lowerand middle-income countries (LMICs) and in animal health settings, where their use in improving diagnostic and treatment decision-making is likely to have the greatest impact. The aim of this study was to evaluate digital CDS tools designed as a direct aid to support diagnosis and/or treatment decisionmaking, by reviewing their scope, functions, methodologies, and quality. Recommendations for the development of veterinary CDS tools in LMICs are then provided.

**Methods:**

The review considered studies and reports published between January 2017 and October 2023 in the English language in peer-reviewed and gray literature.

**Results:**

A total of 41 studies and reports detailing CDS tools were included in the final review, with 35 CDS tools designed for human healthcare settings and six tools for animal healthcare settings. Of the tools reviewed, the majority were deployed in high-income countries (80.5%). Support for AMS programs was a feature in 12 (29.3%) of the tools, with 10 tools in human healthcare settings. The capabilities of the CDS tools varied when reviewed against the GUIDES checklist.

**Discussion:**

We recommend a methodological approach for the development of veterinary CDS tools in LMICs predicated on securing sufficient and sustainable funding. Employing a multidisciplinary development team is an important first step. Developing standalone CDS tools using Bayesian algorithms based on local expert knowledge will provide users with rapid and reliable access to quality guidance on diagnoses and treatments. Such tools are likely to contribute to improved disease management on farms and reduce inappropriate antimicrobial use, thus supporting AMS practices in areas of high need.

## 1 Introduction

To address the global threat of antimicrobial resistance, antimicrobial stewardship (AMS) programs have been implemented in both the human and animal health sectors (1, 2). AMS refers to a coherent set of actions that promote the responsible use of antimicrobials, including optimal selection, dose, route of administration, duration, and control of antibiotic treatment (3–5). These programs play a pivotal role in slowing the development of antimicrobial resistance by promoting responsible and judicious use of antimicrobial agents and preserving their future effectiveness. However, the success of AMS programs often hinges on the support, education, and behavior of those who make prescribing decisions and administer antimicrobial agents (6, 7). Inappropriate or overuse of antimicrobials persists because a significant portion of antimicrobial prescriptions and treatments are overseen by individuals who may possess a limited understanding of the risks associated with antimicrobial resistance (8, 9). While rapid and accurate diagnostic tools are necessary for effective AMS, treatment decisions are often made in the absence of information on the infectious agent and antimicrobial sensitivity profile. This is especially so in resource-limited settings, where treatment decisions frequently result in inappropriate antimicrobial use (10–12). In response to these challenges, digital clinical decision support (CDS) tools have been designed to assist clinicians in making evidence-based diagnostic and therapeutic decisions to enhance AMS at the point-of-care.

Digital CDS tools are of increasing importance in human medicine. They are used in diverse medical sectors and clinical environments, ranging from hospitals to primary healthcare settings, and increasingly for interdisciplinary care of patients with complex needs. CDS tools integrate patient-specific data with clinical knowledge into a computerized “consultation” process to support clinical decision-making (13). These tools offer a wide variety of functions, including interpreting diagnostic test results, aiding in surgical decision-making, and generating prescriptions. CDS tools can also provide additional features such as integration with electronic health records, warning alerts and pop-up reminders on patient encounters, computerized treatment guidelines, automated data entry, and dynamic interactive programs customized for individual patients (14). CDS tools are also increasingly used to assist clinical decision-making in antimicrobial management by facilitating the selection of appropriate antimicrobials and determining the correct dosage, route of administration, and duration of treatment (15, 16). Over time, CDS tools have demonstrated many benefits, including improving diagnostic accuracy, treatment selection, antimicrobial prescription, reducing antimicrobial usage and hospital admissions, shortening hospital stays, and decreasing mortality rates and healthcare costs (17, 18).

The rapid growth of information technology developments has seen “simple” CDS tools evolve to include advanced mathematical modeling such as multivariate regression, neural networks, decision trees, and probabilistic models, such as Bayesian networks (19, 20). These mathematical models can be applied as CDS tool inference engine algorithms to enable the estimation of the likelihood of a patient suffering from a particular disease (informing diagnostic decisions) (21, 22), identification of the optimal treatment strategy for the patient’s condition (informing treatment decisions) (23, 24), and/or estimation of the probability that the selected treatment will result in a certain outcome (informing prognosis estimations) (25, 26). This rapid technological advancement has seen CDS tools transformed from traditional paper-based formats to digital forms, encompassing computer software, websites, or mobile phone applications. They can function as standalone tools or be integrated into complex IT systems, such as hospital electronic health records (27). For example, it has been reported that approximately 40% of US hospitals have adopted CDS integration with electronic health records, alongside other high-income countries such as Canada, the United Kingdom, Denmark, and Australia (28). As health data become increasingly large and complex, new CDS tools require increasingly powerful and sophisticated methodologies, such as artificial intelligence and machine learning. These advanced technologies have the potential to transform diagnosis and therapy at the point of care.

In contrast to human health, CDS tools are less widespread in veterinary settings, although several CDS tools have been reported in the literature for companion animals (29), cattle (30), pigs (31), poultry (32), horses (33), and aquaculture (34, 35). Veterinary CDS tools appear to be primarily developed for sophisticated livestock farming enterprises or for veterinary hospitals equipped with advanced technologies, two scenarios mostly found in high-income countries. However, arguably the greatest potential for veterinary CDS tools lies in those countries where veterinary infrastructure (human resources and diagnostic laboratories) is limited. Rapid diagnosis of the disease is essential to controlling an outbreak, and while it is ideal that veterinarians are available, this is not always possible, especially in remote locations or where there is an absence of veterinary infrastructure. In these cases, having diagnostic support in the form of a CDS tool that can emulate the way a veterinarian thinks could be an aid in managing disease. For example, a study carried out in Ethiopia demonstrated that the use of a smartphone-based application can be a valuable means to provide disease diagnosis and appropriate treatment recommendations by less experienced animal health professionals, which may lead to increased animal productivity (36). Another CDS tool, Fish-VET has been in operation since 1996 and has shown to be a useful diagnostic aid for veterinarians, students, and others seeking to manage disease in tropical and pond fish (34). Properly designed CDS tools have been shown to be able to be used by other animal health professionals, such as paravet or directly by producers. Thus, CDS tools would appear to be valuable for managing disease in livestock populations in remote areas of low- and middle-income countries (LMICs) with limited access to animal healthcare institutions or veterinary professionals (37, 38).

Several publications have reviewed CDS tools, focusing on their features, outputs, benefits, and limitations (39–41), and others have reported on the underlying technologies and methodologies adopted by CDS tools (28, 42–44). Meanwhile, none have explored digital CDS tools that support AMS, and none have explored CDS tool use in the context of animal health in low- and middle-income countries. Moreover, there is limited information regarding CDS tool development in the early design phase, in the construction of mathematical models, or in the evaluation of factors that contribute to the successful implementation of CDS tools. Therefore, our scoping review aims to address these knowledge gaps. Specifically, we aim to (i) identify the scope, functions, and impact of digital CDS tools on diagnosis, treatment, and AMS, (ii) describe the methodologies and technical designs used in the development of these CDS tools, (iii) assess their quality and effectiveness using the GUIDES checklist (45), and (iv) provide recommendations for the development of veterinary CDS tools aimed at enhancing AMS in LMICs.

## 2 Methods

This study was designed as a scoping review to investigate the literature and identify knowledge gaps using a systematic search process. This scoping review was conducted and reported according to the Preferred Reporting Items for Systematic Reviews and Meta-Analyses (PRISMA) guidelines for Scoping Reviews (PRISMA-ScR) (46).

### 2.1 Eligibility criteria

With the definition of CDS tool varies depending on its function and feature, digital CDS tools reviewed in this study were defined as tools designed as a direct aid to support diagnosis and or treatment decision-making by providing real-time output based on computerized clinical knowledge (28). The published scientific literature and gray literature considered for inclusion in this review were those that described any form of a digital CDS tool that was (i) currently implemented and used, or being piloted, or in trial, (ii) intended to support diagnosis and/or treatment, (iii) targeted toward human or animal health, and (iv) published in scientific or gray literature between 1 January 2017 and 24 October 2023. Studies were excluded if the CDS tool was not digital (i.e., not computer software, website, and/or mobile application based), if the tool did not fit within the definition of a digital CDS tool previously described, if the study did not provide sufficient information on the features and functions of the tool (including where full text was not accessible), and/or if the article was not published in English.

### 2.2 Search strategy

PubMed Central was the primary information source for this review and was searched on three occasions in June 2022, January 2023, and October 2023. Google was used as a secondary database to scan for additional studies and gray literature using similar search terms as the primary database. To enhance the electronic database search, Google was also searched for gray literature on CDS tools. Additionally, a “backward snowballing” approach developed by Wohlin (47) was utilized by scanning the reference lists of articles included for extraction and analysis along with gray literature to identify digital CDS tools missed from the primary and secondary search strategies. The search strategy used multiple combinations of keywords relating to the development and implementation of digital CDS tools and CDS systems in human or veterinary health. The final search terms used for PubMed Central were *(“digital tool”[Title/Abstract] OR “digital support tool”[Title/Abstract] OR “decision support tool”[Title/Abstract] OR “decision support system”[Title/Abstract]) AND (“health”[Title/Abstract] OR “veterinary”[Title/Abstract]) AND 2017/01/01: 3000/12/31[Date—Publication]*. Keyword searches used in Google included “*decision support tool” and “decision support system*”; additional words were added at the beginning of the keywords, including “*digital*,” “*diagnosis*,” “*animal*,” and “*veterinary*.” Screening was conducted on the first 100 results of each keyword combination.

### 2.3 Study selection

**Figure 1:**
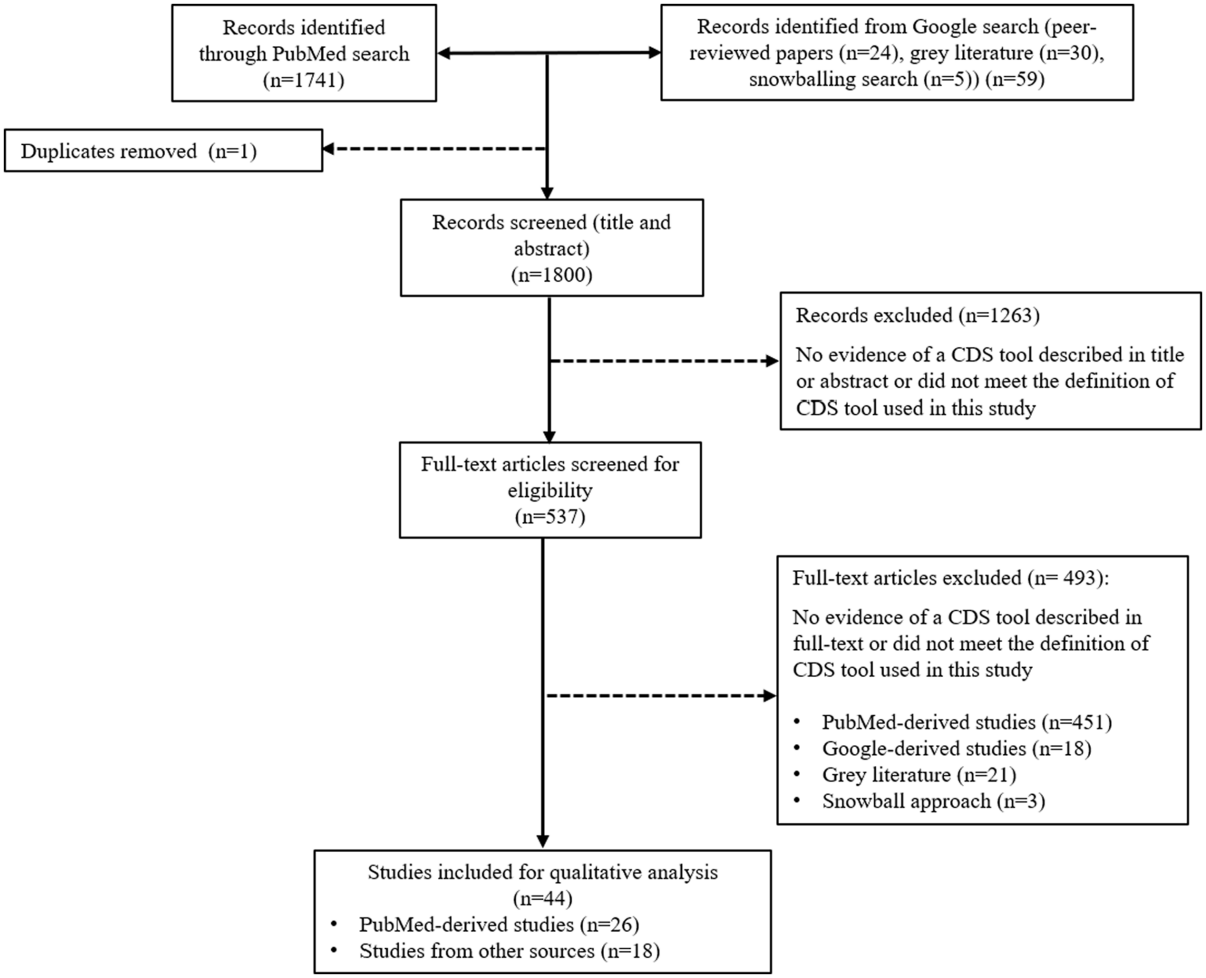
Flow chart mapping out the number of articles identified, screened, and excluded together with reasons for exclusion.

Study selection was performed in February 2023 and October 2023. Titles and abstracts retrieved from each search were exported into Microsoft Excel. Titles and abstracts of identified publications were screened against the eligibility criteria by one of the two reviewers (HY and SB), along with a subset of excluded studies. All potentially relevant publications were independently checked by the second reviewer (HY or SB). Full texts were accessed to assess eligibility and determine inclusion by both reviewers (HY and SB). For gray literature, a full-text assessment was conducted to determine the eligibility of the article to be included in the analysis. Disagreements were resolved mutually (See Figure 1).

### 2.4 Data extraction and analysis

Study characteristics, including CDS type, study design, features or functions of the tool, technical workflow design, and relevant outcomes, were extracted manually into an MS Excel spreadsheet, according to the review’s data collection categories (Table 1). The quality of the CDS tools reviewed in this study was assessed using an adapted version of the GUIDES checklist (45, 49). The checklist consists of four domains that include: (i) CDS context, which refers to the set of conditions for the CDS tool to be potentially successful (ii) CDS content, which refers to the factors that determine the success of output produced by the tool, (iii) CDS system, which refers to the features available on the CDS tool, and (iv) CDS implementation, which refers to the factors influencing how CDS is incorporated into practice setting. Each domain consisted of four success factors, and information on each factor being addressed by each CDS tool is extracted.

**Table 1:** Data collection category for data extraction and charting.

Data category	Data extracted
CDS tool description	Description of the digital CDS tool including the name of the tool, year of development, name, and economic status of the country or countries in which the tool was implemented categorized as: (i) low-income countries (LICs), (ii) lower-middle-income countries (LMICs), (iii) middle-income countries (MICs), and (iv) high-income countries (HICs) (48) Tool purpose(s), target health settings, user groups, and implementation targets^a^
CDS tool methodology	Study timeline Tool form and architecture (database, inference engine, and interface) Mathematical methodology used to develop the CDS tool inference engine Development stages, processes, and the development team Data collection and input Results and recommendations generated by the tool
CDS tool functions and features	CDS tool scope and output Functions relevant to patient safety, clinical management, and cost containment Administrative function and automation Diagnostics support (imaging, laboratory, and pathology) Patient decision support Improvements in documentation of decision-making Workflow improvement
CDS tool benefits, limitations, opportunities, and support to Antimicrobial Stewardship	CDS tool output to support antimicrobial stewardship Benefits generated by CDS tool usage Issues or limitations associated with the tool and its use Opportunities or future directions for how CDS tools can be developed

a Implementation target is the beneficiaries of the CDS tool in human health (e.g., hospital patients, primary-care patients, and pediatric patients) and animal health (e.g., pet animals, pigs, and cattle).

## 3 Results

The primary search resulted in 1,741 articles for the review screening process. On the first screening (title and abstract), 1,263 articles were excluded because they did not meet the definition of a CDS tool specified for this review or were duplicates. On the second screening (full text), 451 articles were excluded, leaving 27 articles within the scope for this review. The Google search added 52 items to the screening process, comprising 25 peer-reviewed publications and 26 gray literature publications in the form of websites containing information about the tool’s scope, functions, and features. Many peer-reviewed publications found during the Google search were not identified in the primary search performed on PubMed Central. We believe this was related to the large number of errors reported on information retrieval in PubMed (50, 51), due to a lack of standardization and formatting of the articles, including title and abstract, inconsistent terminology to describe CDS tools (52), and incorrect indexing (53). The gray literature included in the screening process consisted of commercially available CDS tools (*n* = 23), government-funded CDS tools (*n* = 2), not-for-profit development projects (*n* = 2), a university institution tool (*n* = 2), and a CDS tool developed from a collaboration between a university and private company (*n* = 1). Out of 30 gray literature, 8 were included in the analysis, consisting of 7 commercially available CDS tools and 1 CDS tool developed by the university. The snowballing approach added a further five peer-reviewed scientific publications. Publications included from snowballing were not captured on the primary and secondary searches because the title or abstract did not contain the search query keywords (for example, an article used the name of the tool). Of the 57 items from secondary searches, 41 were ineligible for inclusion, leaving 16 items for inclusion. Thus, for the review, a total of 44 CDS tools were included for data extraction and analysis, comprising 36 peer-reviewed publications, 7 commercial CDS tools, and 1 free-access CDS tool developed by the university. The final 44 CDS tools are summarized in Supplementary Table S1.

The majority of CDS tools evaluated were designed for human health (*n* = 35), in addition to a number of CDS tools for animal health (*n* = 9) (Table 2). Of the CDS tools for humans, 13 targeted general patients in hospitals (15, 17, 18, 54–57), healthcare facilities (21, 36, 58–60), or primary healthcare (61), while 5 tools targeted pediatric patients (23, 26, 62–64) (Table 2). The remaining CDS tools assisted with decision-making for pregnancy (65), diabetes (66), critically ill patients (67), older adult patients with polypharmacy (68), patients with syncope (69), drug allergies (25), diarrhea (70), neurological disorders (71), urinary tract infections (72), dermatology symptoms (22), obstructive sleep apnea (73), hypertension (74), systemic lupus erythematosus (75), as well as patients requiring joint replacements (76) and oncology surgery (77). The CDS tools for animals were for cattle (36, 80, 81), swine (31, 85), companion animals (29, 82, 83), and a CDS tool for dogs, cats, cattle, horses, sheep, goats, swine, birds, and poultry (84). Eleven digital CDS tools were commercially available, all implemented in high-income countries (15, 18, 22, 29, 31, 58, 59, 62, 82, 83, 85). Of the commercially available CDS tools, six were for humans (15, 18, 22, 58, 59, 62) and five for animals (22, 29, 31, 82, 83). A subscription fee was required for three human CDS tools (15, 22, 58) and four veterinary tools (29, 31, 82, 83). Comprehensive information on each digital CDS tool is available in Supplementary Table S1.

**Table 2:** Summary of digital CDS tool baseline information.

Summary items	No. tools (%)	References
Target of CDS tool use		
Human	35	(15, 17, 18, 21–26, 54–79)
Animal	9	(29, 31, 36, 80–84)
Target setting of CDS tool use		
Hospital	20	(15, 17, 18, 23–25, 54–57, 62, 63, 67, 69, 70, 73, 74, 76–78)
Healthcare facilities^a^	9	(21, 22, 58–60, 64, 71, 72, 79)
Farm	5	(31, 36, 80, 81, 85)
Primary healthcare	3	(61, 65, 66)
Animal health clinic and hospital	4	(29, 82–84)
Primary school	1	(26)
Not specified	2	(68, 75)
User of CDS tool		
Clinician^b^	14	(18, 21, 22, 24, 26, 62–65, 68–70, 76, 79)
Physician^c^	10	(17, 55, 56, 60, 61, 67, 72–74, 77)
Healthcare professional^d^	7	(15, 23, 25, 57–59, 75)
Clinicians and patient	3	(66, 71, 78)
Veterinarian	8	(29, 36, 80–85)
Farm worker and veterinarian	1	(31)
Pharmacist	1	(54)
Form of CDS tool		
Computer software	16	(17, 18, 23, 24, 26, 54, 56, 57, 61–63, 67, 68, 72, 73, 79)
Mobile health application	9	(15, 25, 31, 36, 59, 64, 65, 69, 70)
Web-based	12	(21, 22, 29, 60, 66, 74, 75, 77, 78, 80, 84, 85)
Mobile health applications and web-based	6	(55, 58, 71, 76, 81, 82)
Computer software and mobile health application	1 (2.3%)	(83)
CDS tool version		
Standalone	28	(10, 17, 19, 20, 24, 26, 31, 49, 50, 53, 54, 58, 59, 63–65, 67–75, 77, 81, 85)
Integrated in hospital electronic health record	13	(17, 23, 26, 54, 57, 58, 61–63, 66–68, 72)
Standalone and integrated	2	(18, 83)
Not specified	1	(21)
Phase of CDS tool		
Development	24	(17, 21, 23–25, 54, 56, 57, 59, 60, 62, 65, 66, 68, 69, 73–81)
Implementation	14	(15, 18, 22, 29, 31, 36, 58, 61, 70, 71, 82–85)
Development and implementation	6	(26, 55, 63, 64, 67, 72)
Level of Application		
International	11	(15, 22, 29, 58, 59, 64, 65, 82–85)
National	10	(17, 18, 31, 36, 54, 57, 61, 62, 71, 76)
District	1	(70)
Facility	9	(21, 25, 55, 56, 63, 67, 72, 73, 77)
Not specified	13	(23, 24, 26, 60, 66, 68, 69, 74, 75, 78–81)
Antimicrobial stewardship support		
Yes	12 12	(15, 17, 18, 29, 31, 54, 58, 62, 64, 70, 72, 79)
No	28	(21, 22, 24, 26, 55–57, 59, 60, 63, 65–69, 71, 73–78, 80–85)
Indirectly	4	(23, 25, 36, 61)

a The target setting of CDS tools including multi-institutions at different levels, e.g., hospitals, primary healthcare, and clinic.

b Clinicians refer to medical professionals (doctors, nurses, or other medical professionals) who work with patients to diagnose and treat health conditions.

c Physicians refer to doctors who diagnose and treat a patient’s illness and may specialize in a particular area of medicine (e.g., surgery, internist, and pediatric).

d Healthcare professional including physicians, clinicians, nurses, and pharmacists.

**Figure 2:**
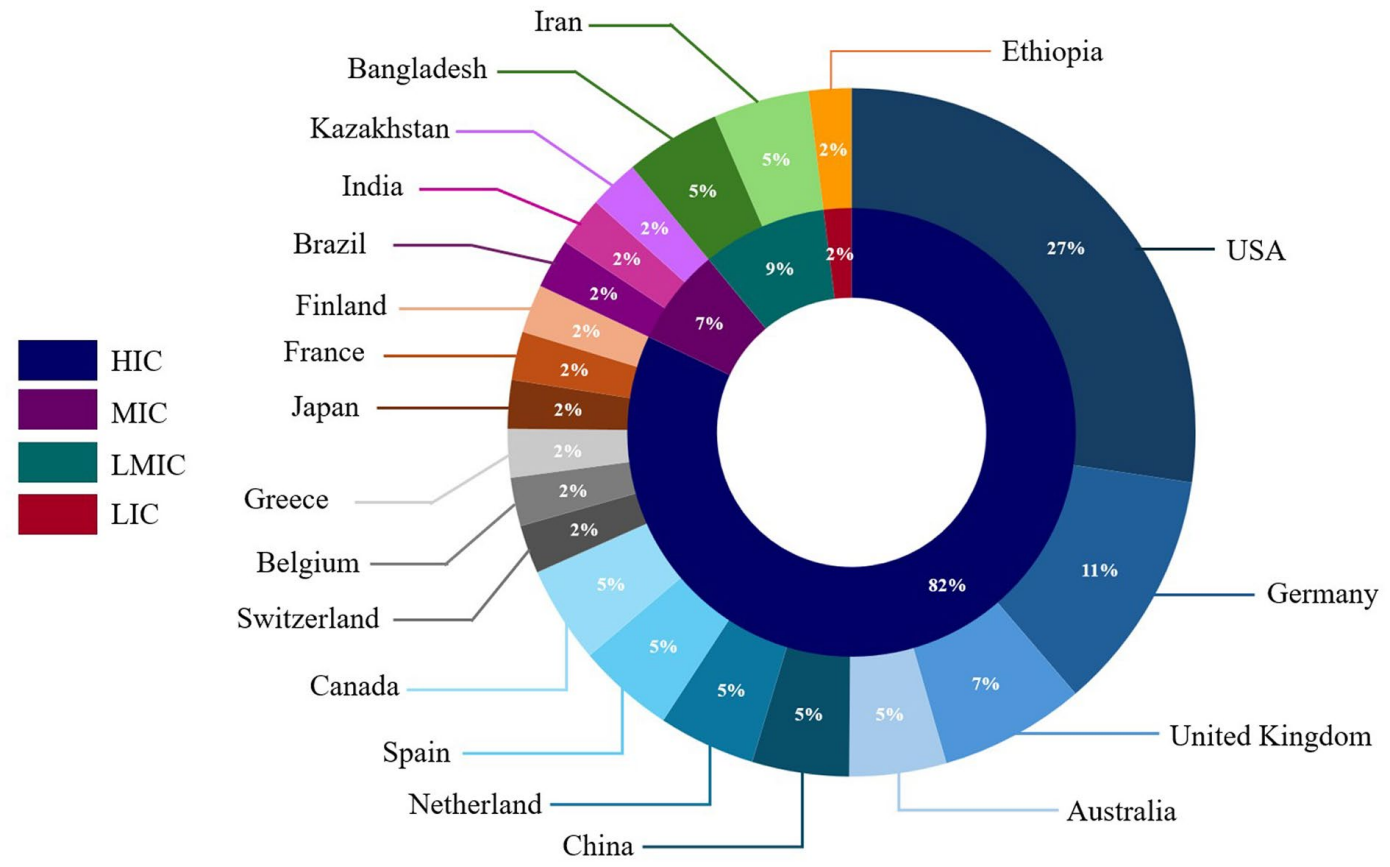
Distribution of clinical decision support tools included in the scoping review by country or origin and country economic status.

Most CDS tools reviewed were developed or implemented in high-income countries (*n* = 36, 82%) (Figure 2). The United States of America had the most CDS tools (*n* = 12, 27%), followed by Germany (*n* = 5, 11%). Among CDS tools developed or implemented in countries with lower economic status, 7% were from middle-income countries, 9% were from lower-middle-income countries, and 2% were from low-income countries (Figure 2). The majority of the CDS tools reviewed (*n* = 28) were standalone CDS tools, while 13 CDS tools were integrated into electronic health records in hospital settings. Two CDS tools had both standalone and integrated features (18, 83).

### 3.1 Digital CDS tool scope and function

The CDS tools reviewed vary in functions, including patient safety, clinical management, cost containment, administrative functions/automation, diagnostics support (including diagnostic imaging, laboratory diagnostics, and pathology), patient decision support, better documentation, and workflow improvement (Table 3). Most of the human and veterinary CDS tools (*n* = 40) have the capacity to improve patient safety by reducing diagnostic and or treatment errors. Twenty-seven CDS tools generate diagnostic output, with treatment output being generated by 25 tools. Seventeen CDS tools can augment and visualize laboratory tests when providing diagnosis or treatment recommendations. Eighteen CDS tools demonstrated cost-effectiveness by reducing unnecessary treatment (15, 17, 18, 23, 25, 66, 70, 71, 76), duration of stay in hospital (69), laboratory tests (59, 83), and health provider workload (31, 36, 54, 55, 58, 82).

**Table 3:** Functions and features of reviewed digital clinical decision support (CDS) tools.

Functions and features	Description	No. tools (%)	References
Patient Safety	Capacity to reduce the probability of diagnosis and treatment/prescription errors	40	(10, 12, 16–20, 24, 26, 31, 46, 47, 49, 51–73, 75, 77, 80, 81)
Clinical management	Promote compliance with clinical guidelines on management, treatment, provide follow-up, and reminders	23	(10, 12, 17, 19, 20, 47, 49, 52, 54, 56, 57, 59, 62–64, 66–69, 71–73, 75, 81)
Cost containment	Reduce laboratory tests performed, avoid order duplication, and suggest more efficient patient management practices and medication or treatment options, to reduce provider workload and time allocation	18	(10, 12, 18, 20, 26, 31, 46, 47, 49, 52, 53, 60, 63–65, 70, 75, 80)
Administrative function/automation	Capacity for automated documentation and auto-fill, diagnostic code selection	19	(10, 12, 18, 24, 26, 46, 49, 51, 54–57, 59, 61, 62, 65, 66, 68, 80)
Diagnostics support	Provide diagnosis suggestions based on patient data and output based on the CDS calculation or test result	27	(16, 17, 20, 21, 24, 31, 49–51, 53, 54, 57–59, 64, 66, 67, 71–73, 75, 77, 80, 81, 85)
Laboratory diagnostic support	Support the extraction, interpretation, and visualization of laboratory test results	17	(10, 17, 18, 21, 24, 46, 51, 53, 54, 57, 60, 61, 64–66, 80, 85)
Treatment support	Provide treatment recommendations for a patient	25	(10, 12, 24, 26, 46, 47, 49, 52–56, 59–62, 64–66, 68–71, 75, 81)
Patient decision support	Functions for the CDS tool to be directly used by patients through personal health records (PHRs) and other systems	7	(18, 22, 59, 62, 71, 78, 79)
Better Documentation	CDS can aggregate data from multiple sources, for example, patient data from electronic health records and laboratory result data	14	(12, 18, 24, 26, 46, 49, 51, 54, 56, 57, 59, 61, 62, 66)
Workflow improvement	Improve the existing clinical workflow through better data submission, sharing, and retrieval to create more effective and faster clinical workflow	21	(12, 18, 19, 24, 26, 46, 49–54, 56, 57, 59, 61–63, 66, 71, 80)

### 3.2 CDS tool development and methodologies

The teams for the development of the digital CDS tools varied in terms of the skills and numbers of contributors. Thirty CDS tools were developed by a multidisciplinary team consisting of people from medical, pharmacy, and/or veterinary backgrounds collaborating with people from computer engineering, economics, and private companies. Four CDS tools were developed by a team of people of varying medical backgrounds, e.g., intensive care medicine, emergency medicine, pediatric medicine, infectious disease, cardiovascular medicine, college of medicine, and department of pathology (57, 64, 67, 69). One digital CDS tool was developed by a team of medical engineers (21). In terms of funding sources for the tool development, 12 tools reviewed in this study were funded by a research grant (24, 36, 55–57, 61, 62, 67, 73, 76–78), 10 by commercial companies (15, 22, 29, 31, 58, 66, 71, 82, 83), 8 by a development project grant (17, 23, 64, 69, 70, 74, 75, 80), and 4 were funded by more than 1 funding source, such as awards and foundation grants (25), foundation and research grants (65, 79) and collaboration of medical research from two countries (59).

The stage of CDS tool development varied in the reviewed papers. Of the 44 studies reviewed, 32 present outcomes from preliminary work into their CDS tool design, such as a literature review, guidelines review, or expert elicitation process (Table 4). Twenty-five studies detail the development of the CDS tool database layer in the form of a computer hardware system or cloud computing, which is used to house data inputs (Table 4). Thirty-one studies describe the development of an inference engine, which is used to determine if CDS conditions have been met and execute queries. Of all the studies reviewed, 25 describe knowledge-based CDS, in which the inference engines run the built-in logic using if-then rules generated from guidelines or expert elicitation and 15 describe non-knowledge-based CDS, in which the engine applies machine learning of mathematical models such as genetic algorithm and artificial neural networks (86). Thirty-one CDS tools provide information on the user interface of the tool for data input from the patient toward the visualization of the results in the form of graphs and charts for the user. Of the 36 published studies evaluated, 20 detailed the study design during the development and/or implementation of the CDS tools. Seven studies used a retrospective study design (15, 25, 56, 59, 62, 71, 76), four studies conducted a pilot study (26, 36, 63, 72), two conducted a usability evaluation (74, 75), and two conducted a prospective observational cohort study (57, 64). The remaining published studies examined in this scoping review detailed cross-sectional (61), interrupted time series study (18), intervention study (70), longitudinal (55), and randomized control trial study designs (67).

**Table 4:** Evaluation of CDS tool development stage.

Level of reporting	No. tools	References
Preliminary work reported such as literature research, guideline review, and expert opinion elicitation as the starting point of development	32	(12, 16, 18–21, 31, 46, 49, 50, 54, 55, 57–59, 61–64, 66–73, 75, 77, 80, 81, 85)
Development of a data management layer for database, storage, and data backing in the computer system or cloud observed	25	(12, 16, 19–21, 46, 49, 51, 56–63, 65, 67, 68, 70–73, 81, 85)
The construction inference engine uses knowledge base datasets of applied rules or construction of algorithms using the mathematical model	31	(10, 12, 16–21, 24, 31, 46, 49–51, 53–73, 81, 85)
Design of the digital CDS tool interface for users to interact when used in clinical practice	32	(12, 16, 18–21, 46, 49–51, 53, 54, 56–62, 65–73, 75, 81, 85)
Validation study to test the CDS tool performance	22	(16, 18–21, 31, 46, 49, 51, 53, 54, 58, 60, 61, 67, 69–73, 80, 85)

Various mathematical methodologies were utilized for the CDS tools reviewed. Most reported methods included Bayesian theorems (*n* = 7), with two each for decision tree and rules-based (IF/THEN) methods (Table 5). For example, VetAfrica-Ethiopia, a CDS tool to assist in the diagnosis of cattle diseases in Africa, utilizes Bayesian algorithms to estimate the probabilities of diseases based on various presented clinical signs in Ethiopian cattle (36). Meanwhile, Autokinetics uses a self-built clinical dosing algorithm to recommend antibiotic selection and dosage. If the tool advice is being followed or if plasma data are available, the tool will use Bayesian estimation to approximate the true pharmacokinetic profile and forecasted pharmacokinetic profile (67). A combination of regression models was utilized by LHSpred (78) to calculate the computed tomography severity score (CTSS) for COVID-19 diagnosis. The “Tool to Reduce Inappropriate Medications” (TRIM), designed to prevent polypharmacy in older adults, utilizes a rules-based algorithm to assess the medication’s appropriateness (68). Details of various mathematical methodologies used by the CDS tools identified in this review are presented in Table 5.

**Table 5:** Mathematical methodologies utilized in the reviewed CDS tools (*n* = 24).

Inference engine methodology	Count (n)	Reference
Bayesian	7	(21, 36, 62, 64, 71, 77, 81)
Decision tree	2	(55, 65)
Rules-based (IF/THEN)	2	(68, 75)
Lasso logistic regression	1	(59)
Logistic regression	1	(69)
Neural network	1	(56)
Minimum mean square error estimation	1	(76)
Logical step	1	(54)
Hierarchical conceptual schema	1	(79)
Business process and model notation (BPMN)	1	(24)
Clinical dosing algorithm and Bayesian	1	(67)
Non-linear least square Curve-stripping Levenberg Marquardt’s Nested logic functions Superposition principle Decision tree	1	(23)
Open-Source Business Rule Management System (BRMS) Drools Drools Expert (rule engine) Drools Fusion	1	(63)
Support Vector Regression (SVR) Multi-Layer Perceptron Regression (MLPR)	1	(78)
Bagged trees algorithm Mamdani-type fuzzy inference system	1	(73)
Machine learning (IBS Watson augmented artificial intelligence system)	1	(82)

### 3.3 CDS tools quality assessment

Of 16 success factors included in the GUIDES checklist, 14 were used to evaluate the CDS tools included in this study. Two factors, namely, the amount of decision support manageable for the target user and governance of the CDS implementation, were excluded as limited evidence was available to assess the CDS tools against these criteria. Many of the included studies and reports describe features that were evaluated in at least one domain, most commonly the CDS context. For some studies, inferences were made to evaluate the quality of the tool where it was not explicitly stated. While most studies describe the context for the CDS tool, fewer provide sufficient information to assess its content, system design, and implementation. Despite the limited information, CDS tool content quality appeared to improve diagnosis decision-making compared to other diagnosis methods, retrospective data of laboratory confirmation, or without the use of the CDS tool. CDS tools also appeared to improve treatment quality compared to other options, including (i) treatment generated without the use of CDS tools (15, 18, 61, 71), (ii) treatment provided by clinicians (62, 70), and (iii) treatment provided by previous tools (66, 67). Details on the CDS tool quality assessment are provided in Table 6.

**Table 6:** CDS tool quality assessment adapted from the GUIDES checklist (45).

Quality domain	Quality factor	CDS assessment	References
CDS context	CDS can achieve the defined quality objectives	44 CDS tools provided evidence that explained the issue in the healthcare setting it aimed to support	(10, 12, 16–21, 24, 26, 31, 46, 47, 49, 50, 52–61, 63–66, 70, 71, 75, 80, 81, 85)
	The quality of the patient data is adequate	41 CDS tools explained that structured patient data were required for the CDS tool analytic processes	(12, 16–21, 24, 26, 31, 46, 49, 50, 52–54, 56–61, 63–66, 70, 71, 74, 75, 77, 80, 81, 85)
	Stakeholders and users accept CDS	15 CDS tools demonstrated clear benefits to the users by improving diagnosis and treatment quality	(10, 17, 47, 49, 50, 55, 56, 60, 61, 64–66, 81)
	CDS can be added to the existing workload, workflows, and systems	14 CDS tools can integrate with hospital electronic health records and to support the clinical workflow	(12, 18, 21, 46, 47, 52, 55–57, 60, 61, 66)
CDS content	The content provides trustworthy evidence-based information	29 CDS tools were developed based on published guidelines and or from renowned expert elicitation	(10, 16, 17, 19–21, 24, 46, 49, 50, 54, 55, 57, 59, 61, 64, 66, 70, 71, 75, 77, 80, 81)
	The decision support is relevant and accurate	15 CDS tools provide information on the accuracy of the tool	(16, 18, 20, 31, 49, 50, 53, 56–58, 60, 61, 63, 81, 85)
	The decision support provides an appropriate call to action	32 CDS tools generated clear recommended actions to the user for diagnosis, patient triage, or treatment decision	(10, 12, 16–20, 24, 26, 49, 52–56, 59–71, 73–75, 80)
CDS system	The system is easy to use	11 CDS tools provide acceptance testing results conducted with the user, one of the objectives of the test was to indicate the tool’s ease of use	(23, 24, 54, 58, 61, 68, 70, 71, 76, 80, 82)
	The decision support is well-delivered	37 CDS tools described the user interface display to ensure that the information provided to users is noticed and easy to process	(12, 16–21, 24, 26, 46, 49–54, 56–62, 65–75, 80, 81, 85)
	The system delivers the decision support to the right target person	All CDS tools reviewed in this study described different types of users according to the tool objective and stage of clinical workflow	(10, 12, 16–21, 24, 26, 31, 46, 47, 49–75, 77, 80, 81, 85)
	The decision support is available at the right time	20 CDS tool in the implementation phase generates immediate outcomes for the user	(10, 17, 21, 24, 26, 31, 47, 49, 52, 55, 57, 58, 61, 64–66, 74, 75, 77, 80)
CDS implementation	Information to users about the CDS system and its functions is appropriate	30 CDS tools provide a communication package that explains features, functions, or how to use the tool	(10, 12, 17, 18, 20, 24, 26, 46, 49, 51–57, 59, 60, 62, 64–66, 69–71, 74, 75, 80, 81, 85)
	Other barriers and facilitators to compliance with the decision support advice are assessed/addressed	22 CDS tools underwent a validation test and the results generated by the tools were adherent to the guidelines or comparable to the expert	(16, 18–21, 31, 46, 49, 51, 53, 54, 58, 60, 61, 67, 69–73, 75, 85)
	Implementation is stepwise and the improvements in the CDS system are continuous	30 CDS tools in the development stage explained the stepwise approach the tool development to the point of user readiness and the strategy for pilot or implementation	(12, 16, 18–21, 46, 49–51, 53, 54, 56–63, 66–73, 81, 85)

### 3.4 Antimicrobial stewardship

Some CDS tools reviewed in this study are capable of supporting AMS principles (*n* = 12), including two CDS tools designed for animals (Table 2). AMS CDS tools for humans were implemented in hospitals (15, 17, 18, 54, 62, 70), healthcare facilities (36, 58, 64, 72), and primary healthcare (61). The AMS CDS tools primarily targeted clinicians, physicians, and pharmacists. These tools are designed to reduce medication dosing errors (54) or generate recommendations on antimicrobial dosing and duration based on prescribing guidelines, antibiograms, and pathogen information (15). Four CDS tools demonstrated improvement in AMS practices by reducing unnecessary or inappropriate antimicrobial use (15, 18, 70, 72). For example, one study reported that a CDS tool deployed in two academic hospitals in Canada saved $111,296 in antimicrobial expenditure over the 6 months period of implementation (15). Significant changes in antimicrobial use practices were reported in some studies where the CDS tool was designed to relatively increase or decrease the use of specific antimicrobials through its recommendations (18) and provided recommendations for antimicrobial use that were consistent with prescribing guidelines and recommendations (72).

## 4 Discussion

Our review of current CDS tools found that while such tools exist in a range of human healthcare settings, there is a dearth of such tools in veterinary settings. Further, while several CDS tools supported AMS practices in human healthcare settings, few supported AMS practices in animal health, an area of particular concern in the control of antimicrobial resistance (10, 11). The disparity in CDS tool availability based on a country’s economic status is stark, with most tools designed for hospital settings in higher-income countries. Very few CDS tools are available in LMICs, and this is where such tools may have the greatest impact. CDS tools designed for animals are of particular interest to countries with limited veterinary services and where decisions on the diagnosis and treatment of unwell animals are often made by untrained personnel (87). Our findings suggest that the field is ripe for the development of CDS tools across all livestock sectors, particularly those that support AMS practices that optimize antimicrobial use.

### 4.1 CDS tools context

The use of CDS tools in human healthcare settings has undergone rapid evolution and expansion since their first use in the 1980s (28). Technological advances, a desire to mitigate errors in healthcare, and access to funding have driven much of this evolution. High-income countries have been able to make the most of the technology and funding opportunities compared to LMICs (88). The slow progress in the development and adoption of digital CDS tools in LMICs is multifactorial. Factors such as limited funding for health programs, prioritization of resource allocation (89), high costs associated with tool development, subscription fees, and training users (90) are considered blockers to the development of CDS tools in these countries. Additionally, data ownership and protection in high-income countries are significantly better and protected by law (91). In contrast, in LMICs, personal data breach are a major challenge in digital innovation due to infrastructure limitations and insufficient legal protections (92, 93). This situation deters significant private-sector investment in CDS tool development due to the risk of legal repercussions from patient or client data breaches (94). Thus, the development and implementation of CDS tools in LMICs tend to be heavily dependent on support through international grants or agencies (95). Identifying long-term sustainable funding models in countries that could benefit most from using CDS tools should be a key consideration in the early phases of development.

In comparison to the human health sector, the development of CDS tools for animal health has been slow (96), despite the genuine need for such CDS tools in veterinary medicine in LMICs (97). The development of CDS tools in livestock species is situation-specific and no one CDS tool can be applied across species; further, in livestock industries, treating and nursing a sick animal is not always a priority for economic reasons, thus creating barriers to development. This is particularly the case given that investment in this sector is generally focused on equipment, tools, and software products to monitor production parameters (e.g., milk yields and feed conversion ratios), management parameters (e.g., herd structure and feed), and environmental parameters (e.g., temperature and humidity) (98, 99). When CDS tools have been developed for animals, they have focused primarily on livestock species, with the end users being veterinarians (29, 31, 36) or farm workers (31). An example of a highly effective livestock CDS tool we evaluated is *VetAfrica-Ethiopia*, which is used in Africa to improve herd productivity by supporting better diagnostic decisions where the availability of veterinarians and laboratory capacity is scarce (36).

To design effective tools, extensive interactions are required between biological disciplines (e.g., animal production, health, epidemiology, and microbiology) and computer science, economics, and sociology (37). Fifteen digital CDS tools were developed by multidisciplinary teams (e.g., medical and computer engineering). In line with recommendations for a multidisciplinary approach, as an example, the study conducted to develop the AMS Computerized Decision Support Tool in a Swiss hospital strongly recommended the involvement of a team with expertise both in clinical medicine and IT early in the project (17). Clinicians have an extensive knowledge of clinical workflow that can be translated into the architecture of CDS tools and inform the provision of output that is valuable from the user perspective (100). Nonetheless, with multidisciplinary teams comes the risk of miscommunication and misunderstanding (17). To overcome that, a recently published Delphi study highlighted the additional benefit of having hybrid positions: someone who has knowledge in both medical and IT, understands both languages, and is capable of holding the responsibility of translating the language into the multidisciplinary team (101).

The benefits of CDS tools in clinical practice should be balanced by an understanding of the well-described limitations of these tools. In any situation where disease diagnosis is required, a CDS tool can only suggest plausible diagnoses with an accuracy that reflects the accuracy of the information it receives (35). Like a human expert relying on their clinical judgment, there is always a degree of uncertainty with any outputs from a CDS tool; in some cases, CDS tool even has different sensitivity and specificity for each disease, which must be understood by the user when interpreting those outputs (21, 35, 102). Other challenges limiting the utility of CDS tools in healthcare settings include having tools that require information outside of their workflow, causing alert fatigue due to excessive amount of alerts that are often inconsequential, requiring upskilling and technological aptitude, being limited by poor data quality or “black box” scenarios which lower confidence in outputs, entail high costs, and so on (28). However, the potential positive impacts of CDS tools should outweigh these limitations, many of which could be overcome by proper planning, engagement with stakeholders, and good design. Certainly, in animal health, CDS tools can help unskilled animal careers make informed decisions and take precautionary actions when managing disease outbreaks in the absence of diagnostic tools or veterinary support. In resource-limited settings, even when the CDS tool is unable to make diagnosis, it is still capable of determining if there is a significant risk that the disease is important, thus directing the user and available resources toward only the most important cases.

### 4.2 Development of digital CDS tool

Referring to the GUIDES checklist on the factors that determine the successful implementation of a CDS tool (45), most factors need to be addressed from the development stage. These start with the basic architectural design of a CDS tool, which is comprised of three layers: (i) a database to capture data, (ii) an inference engine that performs data processing, and (iii) a user interface (103). Nineteen CDS tools reviewed in this study reported database development, indicating a crucial step in the early phase of tool development. The database is often a relational database; this should be designed with an appropriate level of performance, security, availability, manageability, and accessibility. Often, the relational database is programmed with a standardized language, for example, Structural Query Language (SQL), to retrieve, store, and manipulate the database and its contents (104). Database maintenance is an important element of tool operationalization (105). In human healthcare CDS tools, 11 studies reported CDS tools that shared a database with electronic health record systems (17, 23, 26, 54, 58, 61–63, 66, 67, 72). Using an existing, well-maintained, and functional database such as an electronic health record system allowed some of these tools (23, 26, 54, 61, 67) to provide real-time patient alerts and recommendations at the moment the prescribing choice is made, without the need for any intervention (17, 106).

The Bayesian algorithm was one of the most common inference engines (*n* = 7) used in the CDS tools evaluated, comprised of four naïve Bayesian classifiers and three Bayesian networks. Naïve Bayesian classifiers are relatively simple probabilistic classification algorithms that are computationally efficient (96). Naïve Bayesian classifiers assume that all input attributes are independent of each other and are shown to be optimal in minimizing misclassification in outcomes. Thus, reducing the need for an expert in pathogenesis to establish causal dependencies, as required in traditional Bayesian algorithms (19, 107). Studies have shown the naive Bayesian classifier model performs well when compared to more complex classification models, even if assumptions of independence of input attributes are violated (108–110). For example, the CPT tool was designed with a naïve Bayesian algorithm to support physicians with the treatment of oncologic disease using laryngeal cancer (LC), and the prototype provided correct model predictions in all cases throughout the validation (77). In veterinary settings, there is a desire to have CDS tools capable of aiding in the diagnosis of many diseases. Bayesian classifier models are particularly appealing for this purpose, given their flexibility compared to rules-based models (102), given the complexities involved in diagnosing diseases in animals. These complexities include the similarity of clinical signs among many diseases, variations in how animals within a population express clinical signs, changes in clinical signs as a disease progresses, and the potential for animals to suffer from more than one disease simultaneously.

However, Bayesian algorithms are not without some disadvantages. For example, McKendrick et al. highlight that the logical processing of Bayesian algorithms is not clear to users, as data inputted into the system are processed using a mathematical structure unlike rules-based algorithms (102). Additionally, Bayesian algorithms process all available information each time a user adds new data to the system, and so the new data are analyzed in the context of the previous information. In the case of Bayesian networks, while it can model complex problems where there is a significant degree of uncertainty and causal dependencies are involved, they require intensive computational development, limiting their practicality in a big data setting (19, 55). Despite these limitations, a significant benefit of Bayesian models as CDS tools in animal health is their potential for application in other livestock sectors. Once the investment in designing the algorithm and software has been made, they can be utilized in other sectors, provided conditional probabilities for that sector are available (102). As an example of the use of a Bayesian algorithm, the *VetAfrica-Ethiopia* CDS tool assists clinical diagnosis of cattle disease in a resource-limited setting and has demonstrated benefits in assisting less experienced animal health professionals despite having low accuracy for some diseases (36).

There are two categories of supervised machine learning models that currently exist: the white-box and black-box models. Bayesian networks, decision trees, and rule-based (IF/THEN) models are categorized as white-box model and characterized by having lower predictive performance compared to black-box model. However, white-box models possess excellent interpretability since the calculation at each point can be explained (55). In contrast to Bayesian, the decision tree is recognized for its user-friendly structure at the point of practice that allows the underlying logic to be seen by the user. Decision trees are closely related to rule-based models, as the hierarchical data structure of nodes from the root to a leaf in the decision tree corresponds to a rule. The advantage of decision trees over a rule-based model is that it is easier to grasp the model globally because of the tree structure compared to a sequential structure (55, 96). However, the training decision tree classifier is quite complex and potentially spiraling out of control due to the number of nodes in some cases, while a small training set also does not work very well. In addition, only one attribute is tested at a time, consuming a lot of time and being computationally expensive for certain domains (111, 112). On the other hand, black-box models, such as artificial neural networks, possess excellent predictive performance despite having minimal to zero interpretability (55). A study to develop an artificial neural network-based CDS tool for COVID-19 can offer high specificity and good sensitivity. The tool, however, developed through a small population in a single center and had the probability of overfitting problems. Thus, obtaining a multicenter dataset will allow external validation to improve the tool (56).

Validation of the CDS tool is a critical step in the design process. In the tools evaluated in this review, approximately half reported that a validation process was undertaken, with two studies describing the validation study in detail (25, 64). Validation in this review refers to a study in which the developer compares the accuracy of the CDS tool with a “gold standard” measure such as a laboratory test result (97). As most of the tools in this review were categorized as novel tool in their respective field, the availability of a so-called gold standard test was limited. Thus, most of the studies reported that they undertook internal validation using retrospective data from hospital records or publicly available data, and some also undertook external validation with experts. None of the studies reporting on CDS tools for animals reported on a validation study. This may be because any validation exercise using laboratory tests as the gold standard is costly and few clinical records are available for livestock. However, CDS tools will show their benefit if the diagnosis output of a particular disease is comparable to the laboratory test gold standard (28). For example, when the sensitivity and specificity between the CDS tool and laboratory standards are not significantly different (56, 78). Approaches to validation of CDS tools in animals are an important area requiring further consideration.

Several limitations were found in the reviewed CDS tools, mainly associated with the missing validation to provide evidence of tool accuracy (23, 54, 60, 65, 74). Fifteen CDS tools provide information on the tool’s accuracy, which corresponds to the sensitivity and specificity of the tool. However, positive predictive value (PPV) and negative predictive value (NPV) are more relevant when people or animals are being screened (113). Unfortunately, there was limited evidence to extract positive PPV–NPV from the study. As most of the tools are still in the development stage, limited data used for the trial and pilot, and the lack of sensitivity, specificity, PPV, NPV, and validation test might affect the accuracy of tool output when facing complex clinical situations during implementation (23, 25, 59). In addition, several tools also mentioned difficulties in capturing data on the output of the CDS tool compared to the subsequent action taken by the user, as well as the outcome of the action, to quantify the impact of the tool (54, 60, 65). There is also the need for user acceptability testing to ensure the user understands the context-specific information of the tool (24, 54). For CDS tools intended to aid diagnosis, the plausibility/accuracy of suggested diagnoses will only be as good as the base knowledge embedded into the inference engine (56, 77) and information inputted by the user (66, 79). CDS tools are not capable of giving a definitive diagnosis because there will always be a level of uncertainty with the outputs. However, these limitations should not detract from their potentially considerable positive impact on disease management. For situations where a veterinarian is not available, CDS tools can help people take informed action much sooner than they may have in the absence of informed guidance from the tool.

### 4.3 Impact on diagnosis and treatment

In our review, we found evidence that CDS tools can improve the quality of diagnosis of COVID-19 (56, 59), general diseases (79), and respiratory diseases (21). There is great potential for well-designed CDS tools to reduce diagnostic errors, particularly in primary healthcare and resource-limited settings. The CDS tools evaluated demonstrated they could automatically extract laboratory test results (59, 63, 66, 67, 71, 72), including diagnostic imaging (15, 23, 60) and pathology (22, 29) into the CDS tool to improve diagnostic accuracy. In addition to enhanced diagnosis decision-making, four CDS tools demonstrated that users made better treatment decisions, including regarding antimicrobial use, compared to the previous clinical practice tools (15, 18, 62, 67). An additional notable feature of a CDS tool was the generation of a list of treatment options with the prognosis of each option for the user to review before making treatment decisions (71). CDS tools were also shown to be successful in reducing prescribing errors, dosing errors, and contraindications when designed to have safeguards for dosing, duplication of therapies, and drug–drug interactions (61, 71). This is possible because the tools are equipped with features such as automated warnings and drug-event monitoring (114, 115). Among the limited number of CDS tools available for animal health, two CDS tools have a diagnosis function (36, 85), one has a treatment function (31), and one tool has both (29). However, no studies of tools in animal health settings have demonstrated improvement in diagnosis and treatment output.

It has been demonstrated that conventional clinical guidelines for diagnosis and treatment pathways are challenging to put into practice, with low clinical adherence (116, 117). One study suggested that up to 65% of hospital inpatients can be exposed to one or more potentially harmful combinations of medications (118). Meanwhile, CDS tools are able to process complex information and provide patient-specific treatment recommendations, given the probability of the diagnosis, which can inform more appropriate and effective prescription of medications (including the use of antimicrobials) (17, 54, 62, 70, 77). In addition, CDS tools can have features to alert clinicians when a patient has not adhered to a management plan or needs to be followed up, including reminders for testing when particular procedures for the patients need to be applied (119, 120). CDS tools that improve diagnostic accuracy and treatment of disease may be particularly valuable in locations where there is a lack of or limited access to clinical experts (121).

### 4.4 Impact on antimicrobial stewardship

Of the CDS tools designed to improve AMS practices, most were targeted at human healthcare professionals. The key objective of these tools was to verify prudent drug prescriptions, reduce inappropriate use of antimicrobials, and achieve healthcare-related cost savings (16). For example, Guidance MS, a commercially available tool, reported an improvement in appropriate antimicrobial use, a decrease in costs associated with prescribing antimicrobials, and a decrease in healthcare-associated *Clostridium difficile* infection rates, without increasing the length of hospital stay or mortality rates (18). A pilot study of a CDS tool developed for Canadian hospitals reported potential antimicrobial cost savings of $111,296 annually, or $403.98 per acute care bed per year, from improving prescribing behaviors, including appropriate dose and duration, and managing patient medication allergies (15). The CDS tools evaluated also reported improved prescribing practices in resource-limited settings (36, 70), demonstrating that these tools can be an effective intervention for AMS. However, other studies reported that the verification of prescriptions was found to be time-consuming and occasionally unpredictable due to human factors (106, 122). Additionally, in human healthcare settings, it is important to tailor AMS interventions to patients who are most at risk for prescription errors to receive priority care (17).

Just two CDS tools identified in this scoping review were designed to improve AMS practices in animals. Given global concerns about antimicrobial use in livestock species, particularly in LMICs (29, 31), this represents a significant opportunity for innovators to develop CDS tools that improve diagnostic and treatment decision-making and, in doing so, improve AMS practices.

### 4.5 A vision for veterinary CDS tools in low- and middle-income countries

Previous studies have evaluated common technologies and methodologies used to develop and implement CDS tools, but they have predominantly focused on human healthcare settings in high-income countries. Consequently, they often overlook significant global health challenges, such as infectious diseases and antimicrobial resistance, which are prevalent in LMICs. This review aimed to fill this gap by providing additional insights into digital CDS tools and the essential quality factors for their successful development, implementation, and application in human and animal health contexts, particularly in LMICs. Additionally, the review delves into the underlying mathematical methodologies and architectures of various formats and versions of CDS tools, offering further insights into designing future veterinary CDS tools tailored for resource-limited settings.

Development of digital innovations such as information systems and CDS in LMIC is often hindered by limited funding and resources, causing the tool to be unable to perform at the optimal level (64). The reliance on international development projects for development often faces a conflict of interest between stakeholders and a limited duration of project funding, making the tools not adequately developed (17). Furthermore, the user and stakeholders often perceive the innovation as a “free” aid, resulting in a lack of eagerness to maintain sustainability (123). Here, sustainability, means the ability to continue supporting a CDS tool after initial funding and technical assistance have ended (124). Planning for the development of the CDS tool is a crucial first step, including analyzing return on investment and ensuring long-term sustainability so that the CDS tool remains relevant to the sector it is targeted for, but unfortunately, reports of such analyses in the literature are rare (28). Without proper planning, it seems inevitable that a CDS tool will not survive beyond the initial design phase. Factors influencing sustainability include the functionality of the tool, the use of the right technology for the tool, training, and support for end users, leadership and governance, and securing finances beyond the design phase (28, 124). Sustainable business models need to be included in the planning phase of tool development, for example, by adding a subscription fee from the user. CDS tools for animals with a subscription fee have been shown to be more sustainably used when designed to provide real-time recommendations to the user. In addition, CDS tool users would be more willing to pay for the service if the tool can show cost-saving benefits (28). The development phase requires extensive infrastructure and human resources with expert knowledge of the sector and knowledge of coding, programming, computer science, and engineering (90). A multidisciplinary team comprising experienced veterinarians, IT developers, and end-users (e.g., para veterinarians, technicians, and farmers) is necessary to overcome important early challenges in CDS tool development in terms of collaboration and communication of concepts relevant to the development of a tool. It is important that concepts of tool functionality and algorithms are transparent and shared early to assess fitness-for-purpose and feasibility (17). Flexibility in the methodology and approach is essential to allow for the tool to adapt to changing diagnostic and or treatment protocols. In addition, effective validation exercises should occur at each development step to ensure the CDS tool fits with the user’s needs (17).

Another important aspect of CDS tool development is ensuring the successful use of CDS implementation. Making use of standardized guides and protocols, such as the GUIDE checklist, during the development and implementation of the CDS tool will likely improve the probability of designing a CDS tool that is fit-for-purpose. In animal health, CDS tools need to be designed with the end user in mind, especially given the unique and challenging situations where the tool will be needed. For example, a standalone CDS tool as a smartphone app that can be used offline will likely be necessary for users in remote locations or where mobile coverage is limited. Providing easy-to-understand information on how to use the tool and troubleshoot problems without the need for onsite training will likely have a positive impact on adoption, and reduce the funding required for implementation (36, 64).

In the absence of patient data to develop a referral database commonly found in animal healthcare in LMICs, veterinary handbooks and guidelines can be utilized to develop the reference data. However, limitations might occur due to country-specific conditions not covered by the guideline. To overcome this, referral databases can be built based on expert knowledge elicitation to ensure the CDS content provides trustworthy, relevant, and accurate evidence-based information. Transparency of this information and the sources is important to build confidence in the tool. With such limitations, the development of non-knowledge-based inference engines using Bayesian algorithms is recommended. Such algorithms are capable of modeling complex information with causal dependencies such as clinical signs of disease and animal management factors (36, 62, 64, 71). Non-knowledge-based systems have been found to offer significantly reduced healthcare costs and stress on medical practitioners in human healthcare settings (86). In LMICs, access to veterinarians and diagnostic tests is often limited, leading technicians and farmers to make diagnoses based on their judgment alone. This can result in misdiagnosis and treatment errors. Therefore, developing a CDS tool that can guide disease diagnosis with more accuracy than their (untrained) judgment alone and is developed through expert elicitation, will likely be perceived positively by the user. In addition, CDS tools that feature treatment recommendations based on the likely diagnosis should be integrated to encourage users to avoid inappropriate and excessive use of antimicrobials in situations where they may not be needed, thus supporting the AMS program.

Ultimately, the successful implementation of veterinary CDS tools for LMICs is dependent on a number of factors, many of which may not be easily addressed. However, the success of CDS tool implementation will be defined by several factors during development that include (i) proper planning in the initial development of the CDS tool, including a sustainable business model beyond the initial funding, (ii) collaborating with multidisciplinary stakeholders from the start to ensure the development of an effective tool, (iii) designing a low-cost tool that can be used in field situations and with minimal technology requirements, and (iv) being transparent and flexible in its outputs, which will, we think, go a long way toward improving the management of diseases on-farm and ultimately the use of antimicrobials in livestock.

### 4.6 Study limitations

Methodological limitations in this review include potential publication bias during the screening process and the analysis of the CDS tool scope and functions due to the nature of studies identified in this study. This review only used PubMed Central for the peer-reviewed publications source, and other search engines may have identified additional CDS tools for inclusion. The Google search keywords we used in this study were limited, as many other combinations of keywords were not sufficiently discriminating. Our search terms were deliberately narrowed to focus on publications where a CDS tool was mentioned in the title or abstract. However, it became apparent during our search that not all publications that described the development of a CDS tool used the terms included in our search strategy. For example, one study used the term “Intelligent System for Solving Problems” when describing a CDS tool developed to diagnose cow disease and assist in treatment decisions (80). Without checking for this publication using other methods, it would have been missed from our review. Thus, the lack of standardization in describing CDS tools in publications is a limitation of this study and also explains why a number of tools included were identified from the secondary search of gray literature or from the reference lists of publications. Further, we did not undertake a search of the Apple or Android App Store applications. In addition, we only searched among literature in the English language, thus excluding CDS tools that may have been specific to non-English language countries. It was challenging to properly assess CDS tool design and features, as some studies did not provide sufficient detail or were not sufficiently advanced to evaluate them. Therefore, in some instances, we made inferences about tool features that may be misclassified. For example, some peer-reviewed articles utilized commercially available CDS tools (15, 18, 59, 62) where the features and functions of the CDS tools were not presented. In addition, some commercially available tools require a subscription fee before gaining access to them. Therefore, the data on these tools were extracted from available websites or video demonstrations. In this case, shortcomings in the description of the CDS tool made it difficult to tabulate the profile, scope, and functions of the CDS tool. It is understandable that some commercially available CDS tool developers draw a line to maintain competition and protect intellectual property when sharing information about their products. However, we recommend that information related to the design and overall effectiveness of CDS tools be made available for open publication, that will allow other developers, especially in LMICs, to observe.

## 5 Conclusion

To support the development of veterinary CDS tools in LMICs, information regarding tool development, the construction of mathematical models, and quality factors determining the successful implementation of the CDS tool is limited. In addition, none of the previous studies addressed CDS tools in the context of animal health and LMICs, in which global health challenges such as infectious disease and antimicrobial resistance remain an issue. This review summarizes current digital CDS tools available in humans and animals to support clinicians by providing easy and rapid access to information required for diagnosis and treatment decisions at the point of care. Factors that determined the quality and effective use of the CD tool were being addressed, including the development phase, structure, methodology, and common factors that can help increase the probability of successful CDS tool implementation. Ensuring sufficient funding for the development and return on investment analysis to ensure long-term sustainability in the planning is a crucial first step. Deploying a multidisciplinary development team will also help design an effective tool. Developers can use the GUIDE checklist to ensure quality factors are addressed during the development stage, to help achieve quality in design, and to facilitate the successful implementation of the tool. Non-knowledge-based CDS tools using Bayesian algorithms and expert opinion-based referral data are recommended in the absence of data sources. In addition, CDS tools available as mobile phone apps were shown to overcome some of the limitations of veterinary professional support often found in LMICs. We believe that CDS tools with the capacity to model complex information such as clinical signs and management factors by assuming all input attributes are independent to generate diagnostic and treatment recommendations are sorely needed in resource-limited settings. The successful development and implementation of veterinary CDS tools in LMICs will likely contribute to improving treatment decisions and minimizing the inappropriate use of antimicrobial agents, with the primary goal of supporting AMS programs.

## Data availability statement

The original contributions presented in the study are included in the article/Supplementary material, further inquiries can be directed to the corresponding author.

## Author contributions

HY: Writing – original draft, Methodology, Formal analysis, Data curation, Conceptualization. AH: Writing – review & editing. AS: Writing – review & editing, Supervision. AC: Writing – review & editing, Supervision, Funding acquisition, Conceptualization. SB: Writing – review & editing, Methodology, Conceptualization.

## Supplementary material

Supplementary Table S1 Final list of CDS tools included in the study.
